# End stage renal disease caused by thromboangiitis obliterans: a case report

**DOI:** 10.1186/s13256-015-0659-8

**Published:** 2015-08-19

**Authors:** Hyo-Jin Yun, Dong-Il Kim, Kyung-Ho Lee, Seong-Joo Lim, Won-Min Hwang, Sung-Ro Yun, Se-Hee Yoon

**Affiliations:** 1 Division of Nephrology, Department of Internal Medicine, Konyang University College of Medicine, 158 Gwanjeo-dong-ro, Seo-gu, Daejeon, 302-718 South Korea; 2 Department of Radiology, Konyang University College of Medicine, Daejeon, South Korea; 3 Konyang University Myunggok Medical Research Institute, Daejeon, South Korea

**Keywords:** End stage renal disease, Infarction, Kidney, Mesenteric ischemia, Thromboangiitis obliterans

## Abstract

**Introduction:**

Thromboangiitis obliterans or Buerger’s disease is a nonatherosclerotic, segmental, inflammatory vasculitis that is strongly associated with tobacco products and commonly affects the small- and medium-sized arteries of the upper and lower extremities. However, the disease can, rarely, involve large central or visceral arteries. We report here the case of end stage renal disease due to renal artery thrombosis caused by thromboangiitis obliterans.

**Case presentation:**

A 51-year-old Korean man who had previously required amputation of both great toes due to thromboangiitis obliterans presented with left flank pain and oliguria. Both his renal arteries were occluded on contrast-enhanced abdominal computed tomography and abdominal angiography. He also had abdominal angina. He had no risk factor of thromboembolism from cardiac origin, atherosclerosis except for tobacco abuse, collagen diseases or hypercoagulable disorders. Renal failure and mesenteric ischemia associated with thromboangiitis obliterans progression was diagnosed.

**Conclusions:**

Renal failure due to renal artery thrombosis and mesenteric ischemia represents an unusual manifestation of thromboangiitis obliterans. But once it occurs, it can be life-threatening. When we care for a patient with thromboangiitis obliterans, we should pay attention to this rare disease course, and encourage cessation of the smoking of tobacco products.

## Introduction

Thromboangiitis obliterans (TAO) or Buerger’s disease is a nonatherosclerotic, segmental, inflammatory vasculitis that is strongly associated with tobacco products and commonly affects the small- and medium-sized arteries of the upper and lower extremities. It was first described and established in the English literature in 1908 as a clinicopathologic entity distinct from atherosclerosis [1]. TAO usually occurs in young male patients and is associated with tobacco consumption; it presents with a highly cellular thrombus with relative sparing of the blood vessel wall and an absence of elevated acute-phase reactants or immunological markers. It is reported that the prevalence of TAO among all patients with peripheral arterial disease is higher in East Asia (16−66%) than Western Europe (0.5−5.6%) [2]. Although it is rare, the disease can involve large central or visceral arteries and cause intestinal ischemia or renal infarction.

We report a case of end stage renal disease caused by renal artery thrombosis and abdominal angina associated with TAO.

## Case presentation

**Fig. 1:**
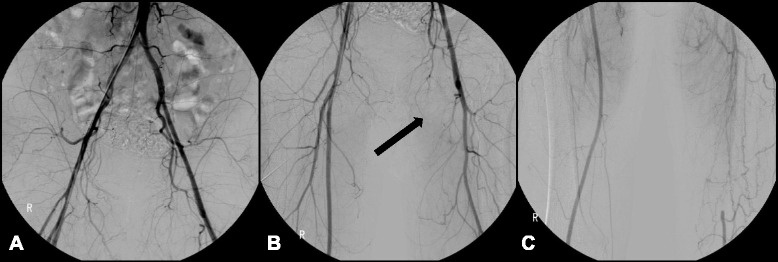
Lower extremity angiography (2004). **a** Both common iliac arteries showed patent flow without stenosis or occlusion. **b** The left superficial femoral artery was occluded at its origin (*arrow*). **c** The left distal superficial femoral artery was reconstituted by an abnormal corkscrew collateral blood flow from the left deep femoral artery

A 51-year-old Korean man was admitted to our hospital because of severe left flank pain, hematuria, and oliguria for 3 days. Additional complaints included epigastric discomfort and generalized weakness, but he denied fever or emesis. He had a medical history of hypertension for 1 year and TAO for 10 years with intermittent claudication. He had undergone amputation of both of his great toes 10 years prior because of gangrenous change due to TAO. At that time, lower extremity angiography showed that the flow of the right distal portion of the popliteal artery and the proximal portion of the tibiofibular artery were remarkably decreased by occlusion. The left superficial femoral artery was also occluded from its origin, at which collateral arteries had developed (Fig. 1). He took beraprost for TAO but had not stopped smoking tobacco products. He had smoked approximately 1 pack per day for 30 years. Four years later, he underwent repeat angiography of his abdominal aorta and lower extremities because of worsening claudication. Occlusion of his left superficial femoral artery, bilateral tibial, and peroneal arteries had progressed. He had never been diagnosed with diabetes mellitus, collagen disease or cardiac disease.

Upon presentation, his blood pressure was 180/100mmHg, and his body temperature was 36.4°C. He complained of severe tenderness in his left costovertebral angle area. Raynaud’s toe, skin nodules and phlebitis were not observed.

Laboratory findings showed the following: white blood cell count (WBC) 9700/uL, hemoglobin (Hb) 12.7g/dL, platelets 201×10^3^/uL, serum creatinine 14.02mg/dL, creatinine clearance 3.6ml/minute/1.73m^2^ according to the Chronic Kidney Disease Epidemiology Collaboration (CKD-EPI) formula, β2 microglobulin 19.80mg/L, phosphorus 5.48mg/dL, intact parathyroid hormone (PTH) 329.5pg/mL, creatine phosphokinase (CPK) 74U/L and lactate dehydrogenase (LDH) 1687IU/L. Urine sediment contained 0 to 2 WBC and 3 to 5 red blood cells (RBC) per field. Urine protein electrophoresis revealed no paraprotein bands. The blood lipid profile, coagulation tests, protein C, protein S activity, complement fractions, antinuclear antibodies, rheumatoid factor, anti-Scl-70, anticardiolipin and antiphospholipid, and antineutrophil cytoplasmic antibodies were all negative or within normal limits. Electrocardiography and echocardiography were normal.

**Fig. 2:**
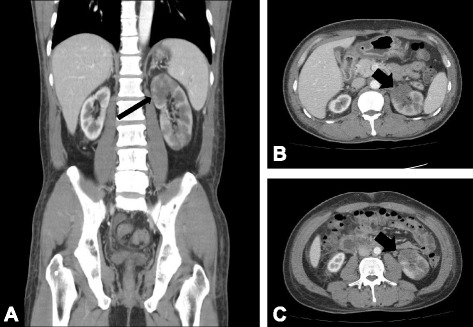
Contrast-enhanced abdominal computed tomography. **a** Coronal and (**b, c**) transverse scans showed left kidney enlargement with a multifocal infarcted area (*arrows*). Neither renal artery was traced from the proximal part on computed tomography

Contrast-enhanced abdominal computed tomography (CT) demonstrated left kidney enlargement (9.3cm) with a multifocal infarcted area and a shrunken right kidney (7.6cm). Neither renal artery was visualized (Fig. 2).

**Fig. 3:**
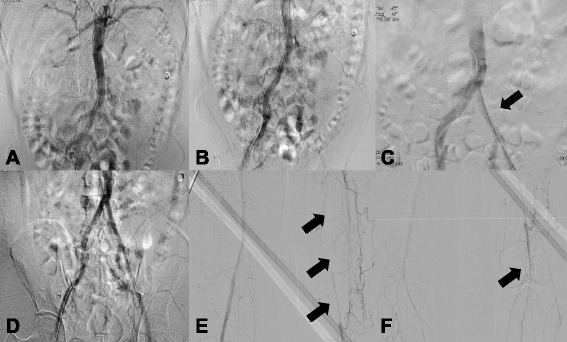
Abdominal and lower extremity angiography (2014). **a** Renal angiography could not identify either renal artery due to total occlusion. **b** Lower extremity angiography showed a chronic total obstruction lesion of the left common iliac artery due to progression of chronic thrombosis. **c** A stent was deployed at the site of occlusion of the left common iliac artery (*arrow*). **d** Flow was recovered. **e** Obstruction of the left superficial femoral artery and abnormal corkscrew collateral blood supply from the left deep femoral artery was similar to that seen in 2004 (*arrows*). **f** The left tibioperoneal trunk was occluded (*arrow*), and blood flow below the knee was supplied by collateral vessels

Abdominal and lower extremity angiography was performed to examine his abdominal aorta and lower extremity arteries. The vascular status in both lower limbs and the viscera had worsened. His superior mesenteric artery, inferior mesenteric artery, both renal arteries, left common iliac artery, and left superficial femoral artery were not visualized, and the arteries below both his knees were occluded. Collateral vessels were well developed in his lower extremities. During examination, a stent was inserted into his left common iliac artery (Fig. 3). Upper extremity angiographic CT showed no abnormal findings. His ankle-brachial index was 0.82 on the right and 0.61 on the left.

**Fig. 4:**
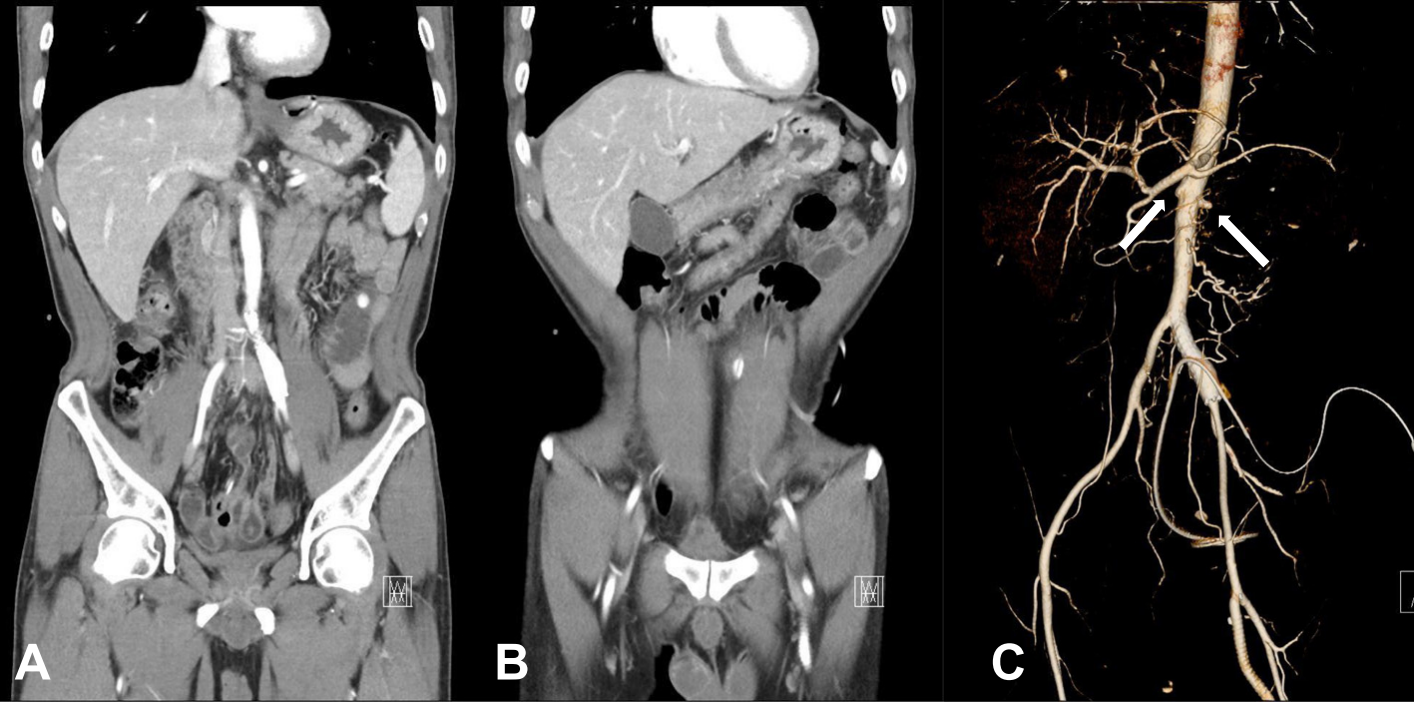
Contrast-enhanced abdominal CT and abdominal aorta CT angiography. Contrast-enhanced abdominal CT demonstrated colitis of the (**a**) hepatic flexure and (**b**) transverse colon, most likely due to ischemic colitis. **c** Abdominal aorta CT angiography showed total occlusion of both renal arteries (*white arrows*). Superior and inferior mesenteric arteries cannot be seen because the arteries were occluded from their origins

Renal failure associated with TAO progression was diagnosed. He started continuous ambulatory peritoneal dialysis (CAPD) and stopped smoking tobacco products. He was discharged with a daily oral anticoagulant, warfarin. Two months after discharge, he complained of postprandial abdominal pain without muscle guarding, preventing him from eating and resulting in an approximately 10kg weight loss. Upper gastrointestinal endoscopy revealed gastric mucosal atrophy; a follow-up contrast-enhanced abdominal CT showed colitis of the hepatic flexure and transverse colon, which was consistent with ischemic colitis. His vessel status had not changed compared with that at the prior examination (Fig. 4). He changed his dialysis modality from CAPD to hemodialysis, which improved his pain. He recently reported abdominal pain with hypotension; however, after a decrease in his hypertension medications and an increase in body weight, his pain resolved.

## Discussion

Many kinds of diseases have been identified as causes of renal infarction, the most common of which are cardiogenic thromboembolism and atheromatous disease. Other less frequent causes include trauma, hypercoagulable state, cocaine abuse, and neoplastic and renal vascular disease [3]. Even though TAO commonly affects the small- and medium-sized arteries of the upper and lower extremities, a few cases of kidney infarction and thrombosis of visceral vessels have been reported [4–9].

In some of these cases, visceral damage is more likely to be a result of atherosclerosis or other diseases, so a broad differential diagnosis is important. The present patient had symptoms typical of TAO. Clinical criteria for the diagnosis of TAO include a cigarette smoking history, onset before age 50, infrapopliteal arterial occlusive disease, either upper limb involvement or phlebitis migrans, and the absence of atherosclerotic risk factors other than cigarette smoking [10]. The present patient had intermittent claudication and an ischemic ulcer beginning at 42 years of age. He had no atherosclerotic risk factors except for tobacco abuse. He denied any drug ingestion other than an antihypertensive agent (nifedipine) and beraprost. Although hypertension was diagnosed 1 year prior, it seemed likely that this was a result of renal artery occlusion caused by TAO. No serologic data suggested collagen disease or anticoagulation disorder. An electrocardiogram did not show atrial fibrillation that could cause distal embolization. An angiogram of his lower limb showed abrupt occlusion and a tree root pattern, findings typical of TAO. Although histological examination of arterial tissue was not possible in the present study, aortography demonstrated no atheromatous plaques in the remaining portion of the aorta. So thrombosis in his visceral arteries including both renal arteries, associated with TAO progression was diagnosed.

We comprehensively reviewed the English literature reporting renal involvement in TAO in either abstract or full text form [4–9]. The clinical characteristics of six patients with TAO who showed renal artery involvement with TAO, including the present case, are summarized in Table 1. Among the seven cases, one was excluded because it was not an English report [7]. The mean age was 40.3 years, and all patients were male. TAO was diagnosed in five of six cases before renal artery involvement, except one in which the medical history could not be found in the literature. The average duration from initial diagnosis of TAO to involvement of the renal arteries was 11 years (range 7–15 years). Severe hypertension was the most common symptom, followed by flank pain caused by renal infarction. In five cases, visceral arteries including the descending aorta were involved. None of the patients stopped smoking tobacco products after they were diagnosed with TAO, highlighting the importance of tobacco smoking cessation to prevent the progression of TAO.

**Table 1:** Reported cases of renal artery involvement of thromboangiitis obliterans

Case	Age/sex	Diagnosed TAO before (duration)	Symptom	Affected visceral artery	Treatment	Reference (reported year)
1	34/M	Yes	Severe hypertension	Right renal artery	Right nephrectomy	Malisoff and Macht (1951) [4]
2	30/M	Yes (15 years)	Diffuse back and muscle pain	Descending aorta, celiac axis, iliac artery, femoral artery, coronary artery, left renal artery		Flesh *et al*. (1977) [5]
3	42/M	Yes (12 years)	Severe hypertension	Left renal artery, aorta below the level of renal artery	Antihypertensive medication	Gomi *et al*. (1978) [6]
4	51/M	Yes	Severe hypertension, respiratory distress	Both renal arteries, descending aorta, celiac trunk, superior mesenteric artery	Hepatorenal artery bypass	Stillaert *et al*. (2003) [8]
5	37/M	Yes (7 years)	Right flank pain, weakness, fever	Intrarenal branches of the right renal artery	Conservative care	Goktas *et al*. (2006) [9]
6	52/M	Yes (10 years)	Left flank pain, anuria, weakness	Descending aorta, both renal arteries, superior mesenteric artery, common iliac artery	Hemodialysis	This case

*M* male, *TAO* thromboangiitis obliterans

In the presented case, the patient complained of postprandial abdominal pain after starting peritoneal dialysis. Intestinal TAO has been rarely reported. Kobayashi *et al*. [11] reported a case of TAO with intestinal ischemia and reviewed the literature. They summarized 26 cases of visceral TAO including their case. The mean age of patients was 39.1, and all but two patients were male. The predominant symptom was abdominal pain, and 20 of 26 patients underwent digestive organ resection. The perioperative mortality rate was 30%, and only three patients underwent conservative treatment. The present patient experienced improvement in postprandial abdominal pain after avoiding dehydration and switching from peritoneal dialysis to hemodialysis. It is known that hemodialysis is more susceptible to intestinal ischemia than peritoneal dialysis because of its more unstable hemodynamics [12]. The patient in this case underwent peritoneal dialysis for this reason; however, even though he was not dehydrated and his blood pressure was not low while receiving peritoneal dialysis, he experienced severe abdominal pain that got worse after starting peritoneal dialysis. His pain was relieved after stopping peritoneal dialysis. We presumed that hyperglycemia of the peritoneal cavity induced several changes, including leukostasis, vasoconstriction, and a pro-inflammatory state that caused aggravation of intestinal hypoxia [13].

## Conclusions

Although visceral involvement, including renal and intestinal arteries, is rare in TAO, once an internal organ is affected, the disease becomes life-threatening and usually cannot be cured. When treating a patient with TAO, we have to carefully observe unusual symptoms such as anuria, flank pain, uncontrolled hypertension, and abdominal pain and strongly encourage cessation of tobacco smoking.

## Consent

Written informed consent was obtained from the patient for the publication of this case report and any accompanying images. A copy of the written consent is available for review by the Editor-in-Chief of this journal.

## Abbreviations

CAPD: Continuous ambulatory peritoneal dialysis
CT: Computed tomography
TAO: Thromboangiitis obliterans
WBC: White blood cell count
